# Modulatory Roles of *AHR*, *FFAR2*, *FXR*, and *TGR5* Gene Expression in Metabolic-Associated Fatty Liver Disease and COVID-19 Outcomes

**DOI:** 10.3390/v16060985

**Published:** 2024-06-19

**Authors:** Mykhailo Buchynskyi, Valentyn Oksenych, Iryna Kamyshna, Ihor Vorobets, Iryna Halabitska, Oleksandr Kamyshnyi

**Affiliations:** 1 Department of Microbiology, Virology, and Immunology, I. Horbachevsky Ternopil National Medical University, 46001 Ternopil, Ukraine; 2 Broegelmann Research Laboratory, Department of Clinical Science, University of Bergen, 5020 Bergen, Norway; 3 Department of Medical Rehabilitation, I. Horbachevsky Ternopil National Medical University, 46001 Ternopil, Ukraine; 4 Ophthalmology Clinic “Vizex”, Naukova St. 96B, 79060 Lviv, Ukraine; 5 Department of Therapy and Family Medicine, I. Horbachevsky Ternopil National Medical University, Voli Square, 1, 46001 Ternopil, Ukraine; halabitska@tdmu.edu.ua

**Keywords:** COVID-19, MAFLD, *AHR*, *FFAR2*, *FXR*, *TGR5*

## Abstract

Metabolic-associated fatty liver disease (MAFLD) is a risk factor for severe COVID-19. This study explores the potential influence of gut hormone receptor and immune response gene expression on COVID-19 outcomes in MAFLD patients. Methods: We investigated gene expression levels of *AHR*, *FFAR2*, *FXR*, and *TGR5* in patients with MAFLD and COVID-19 compared to controls. We examined associations between gene expression and clinical outcomes. Results: COVID-19 patients displayed altered *AHR* expression, potentially impacting immune response and recovery. Downregulated *AHR* in patients with MAFLD correlated with increased coagulation parameters. Elevated *FFAR2* expression in patients with MAFLD was linked to specific immune cell populations and hospital stay duration. A significantly lower *FXR* expression was observed in both MAFLD and severe COVID-19. Conclusion: Our findings suggest potential modulatory roles for *AHR*, *FFAR2*, and *FXR* in COVID-19 and MAFLD.

## 1. Introduction

Since the initial detection of the Severe Acute Respiratory Syndrome Coronavirus 2 (SARS-CoV-2) in late 2019, the virus has had a significant global impact, with over 700 million confirmed cases and 7 million deaths being attributed to COVID-19 [1]. Identifying factors that influence susceptibility to SARS-CoV-2 infection and understanding how pre-existing medical conditions (comorbidities) affect disease severity remain crucial areas of contemporary research [2]. These insights hold significant promise for the development of improved treatment strategies [3,4].

Advanced age, thrombocytopenia (low platelet counts), lymphopenia, and elevated serum concentrations of lactate dehydrogenase, alanine aminotransferase, aspartate aminotransferase, procalcitonin, creatinine, and D-dimer have all been identified as risk factors for severe COVID-19 [5]. In particular, systematic investigations have demonstrated a nearly five-fold increase in the risk of severe illness associated with elevated procalcitonin levels [6]. Furthermore, thrombocytopenia is independently associated with increased morbidity and mortality in patients with COVID-19 [7]. Individuals with primary immunodeficiency are known to be more susceptible to SARS-CoV-2 infection [8].

There is a substantial body of evidence demonstrating a high prevalence of metabolic and vascular disorders among individuals who succumb to COVID-19, with these comorbidities accounting for up to 50% of fatalities [9]. Several studies have specifically highlighted Metabolic Associated Fatty Liver Disease (MAFLD) as a potential independent risk factor for both SARS-CoV-2 acquisition and hospitalization during COVID-19 illness, even after controlling for other aspects of metabolic syndrome [10,11,12]. Furthermore, research suggests that MAFLD may contribute to altered gene expression patterns, potentially influencing the course of COVID-19 [13,14]. To elucidate the intricate interplay between these conditions, further investigation into the differential gene expression patterns associated with co-occurrence of MAFLD and COVID-19 is warranted [15,16].

Within the realm of immunology, the aryl hydrocarbon receptor (*AHR*) is a ligand-activated transcription factor classified as a nuclear receptor [17]. This widely expressed protein is found in both innate and adaptive immune cells. AHR plays a multifaceted role in physiological processes, including the regulation of immune responses, inflammatory pathways, and environmental adaptations [18].

Activation of indoleamine 2,3-dioxygenase 1 (IDO1) by immune cells leads to the release of kynurenine (Kyn), a tryptophan (Trp) metabolite. Functioning as an endogenous ligand, Kyn activates *AHR*, thereby potentially hindering protective immunity [19]. Studies have demonstrated that *AHR* activation suppresses the initiation of influenza virus-specific CD8+ T cells within the lung compartment [20]. Interestingly, metabolomic analyses of patients with COVID-19 have revealed a similar alteration in Trp metabolism, favoring the Kyn pathway [21].

Building on this, Giovannoni et al. (2021) proposed a potentially novel therapeutic approach for SARS-CoV-2 infection: *AHR* modulation [22]. Their research suggests that SARS-CoV-2 infection itself activates *AHR*, potentially promoting viral replication. In dendritic cells (DCs), *AHR* activity down-regulates the expression of Major Histocompatibility Complex II (MHC II) molecules. Furthermore, *AHR* regulates the production of inflammatory cytokines such as IL-6, IL-12, IL-15, and IL-18, which are typically produced during DC differentiation [23].

Continuing the exploration of the *AHR* within the context of COVID-19 and MAFLD, research suggests a potential proviral and profibrotic role for *AHR*. Constitutive activation of *AHR* (CA-*AHR*) has been demonstrated to inhibit mitochondrial β-oxidation, increase adipose triglyceride lipase activity, decrease white adipose tissue fat mass, and promote hepatic oxidative stress [24]. Furthermore, studies employing transgenic mice with constitutively active human *AHR* exposed to a high-fat diet exhibited exacerbated steatosis, highlighting a potential link between *AHR* and hepatic fat accumulation [25]. Conversely, *AHR* knockout (KO) mice displayed improved insulin sensitivity and glucose tolerance on a standard chow diet [26]. Moreover, these *AHR*-deficient mice were protected from high-fat diet-induced steatosis, obesity, and inflammation. Lv et al. (2021) demonstrated that the transcription factor AHR up-regulates ACE2 expression, thereby exacerbating the lung pathology that is present during SARS-CoV-2 infection [27].

The cumulative findings from these studies raise intriguing questions regarding the potential role of *AHR* as a proviral host factor in SARS-CoV-2 replication and MAFLD progression.

Previously classified as orphan receptors, FFARs (free fatty acid receptors) are now recognized as G protein-coupled receptors (GPCRs) that mediate metabolic signaling in response to fatty acid (FA) activation. Obesity, type 2 diabetes mellitus (T2DM), and MAFLD are closely linked conditions, each contributing to the metabolic syndrome phenotype [28].

Among FFARs, *FFAR2* (free fatty acid receptor 2) demonstrates particularly widespread expression in adipocytes, enteroendocrine cells, pancreatic β-cells, and various immune cells such as macrophages and neutrophils [29,30]. This receptor regulates both lipid metabolism and glucose levels through its influence on hormone secretion and inflammatory processes [31]. While *FFAR2* is not directly expressed in hepatocytes, its ability to modulate the intestinal microbiota inflammasome may hold implications for MAFLD progression [32]. This effect is likely attributable to the *FFAR2* pathway’s capacity to suppress inflammatory cytokines and mitigate chronic inflammation, both of which are beneficial for metabolic liver disease [33].

Shifting focus to COVID-19, the cytokine storm observed in this disease is triggered by the release of interleukin-6 (IL-6) from alveolar macrophages [34]. Interestingly, *FFAR2* appears to play a role in regulating probiotic activity, which has been shown to reduce IL-6 levels in COVID-19 patients [35,36].

Further connecting the dots between bile acid metabolism and both COVID-19 and MAFLD, we can explore the roles of Farnesoid-X-receptors (*FXR*) and G protein bile acid-activated receptor 1 (*GPBAR*-1), also known as Takeda G-protein-coupled receptor 5 (*TGR5*). These represent the two most well-characterized receptors within the bile acid-activated receptor (BAR) family [37]. Interestingly, these receptors are also expressed at high levels in cells within the innate immune system, including monocytes/macrophages, dendritic cells (DCs), natural killer (NK) cells, and NKT cells [38,39,40].

Similar to *FXR*, *TGR5* appears to exert counter-regulatory effects on the immune response. The activation of *TGR5* promotes a shift in colonic macrophages from a pro-inflammatory M1 phenotype to an anti-inflammatory M2 phenotype [41]. Furthermore, *TGR5* suppresses the expression of pro-inflammatory cytokines such as IFN-γ, IL-1β, IL-6, and TNF-α, while inducing the production of the anti-inflammatory cytokine IL-10 [42].

Clinical studies have revealed alterations in bile acid metabolism (specifically, the composition of bile acids) in patients with COVID-19 as the disease progresses from non-severe/mild-to-severe stages [43,44]. Intestinal dysbiosis, an imbalance in the gut microbiome, is another common feature observed in COVID-19 patients [45]. The presence of SARS-CoV-2 RNA in fecal samples from infected individuals suggests the virus’s potential to replicate within human enterocytes. This viral replication within the gut may contribute to the disruption of bile acid metabolism and diarrhea, a frequent symptom of COVID-19 [46].

A recent study by Stutz et al. (2022) suggests that elevated levels of fecal secondary bile acids are associated with improved clinical outcomes in patients with COVID-19 [47]. This finding can be explained by the immunosuppressive activity of CD4+ regulatory T cells (Tregs). Deconjugated bile acids appear to influence these Tregs, leading to an increase in their numbers. Additionally, the action of these bile acids on DCs has been shown to reduce their immunostimulatory properties [48].

The cellular entry of SARS-CoV-2 is facilitated by the spike protein binding to the angiotensin-converting enzyme 2 (ACE2) receptor. Interestingly, BAs may influence ACE2 expression in various tissues through their interaction with *FXR* and *GPBAR1* [49]. Brevini et al. demonstrated that biliary organoids cultured in the presence of cholic acid (CDCA), a primary bile acid, expressed ACE2. Conversely, the removal of CDCA from the culture medium resulted in a reduction of ACE2 expression [50]. Similarly, BA regulation of ACE2 via GPBAR1 has also been documented. A study by Biagioli et al. (2022) showed that in vivo activation of GPBAR1 increased the production of glucagon-like peptide-1 (GLP-1) in intestinal L cells, which in turn promoted colonic cell expression of ACE2 [51]. These findings suggest that the potential protective effects of bile acid receptors in patients with COVID-19 may be mediated through the modulation of ACE2 expression alongside influencing the immune response.

For patients with MAFLD, the activation of bile acid receptors appears to play a beneficial role in reducing disease severity. *FXR* activation has been shown to decrease steatosis by inhibiting lipogenesis, reduce chemically induced hepatic inflammation and fibrosis, and maintain intestinal barrier integrity, thereby protecting the liver from inflammatory signals originating from gut bacteria [52,53]. Similarly, *TGR5* activation has been demonstrated to mitigate high-fat diet-induced glucose intolerance, insulin resistance, and inflammation, while also protecting against lipopolysaccharide-induced inflammation [54,55].

Taken together, these findings suggest that *AHR*, *FFAR2*, *FXR*, and *TGR5* may play significant roles in the progression of both COVID-19 and MAFLD. Targeting these genes represents a promising therapeutic strategy that has the potential to improve patient outcomes and reduce disease severity. The current study aims to investigate the specific roles of these genes in the co-morbid course of COVID-19 and MAFLD.

## 2. Materials and Methods

### 2.1. Sample Collection

This investigation recruited participants from Ternopil City Community Hospital No. 1. Following informed consent through a signed statement, blood samples were collected and stored at −80 °C until analysis. All procedures adhered to the Declaration of Helsinki and received ethical approval from the I. Horbachevsky Ternopil National Medical University Ethics Committee (protocol No. 74, dated 13 October 2023).

The study population included individuals of European ancestry (Ukrainian ethnicity) aged between 23 and 86 years. Participants were hospitalized between October 2022 and May 2023. Inclusion criteria comprised confirmed COVID-19 diagnosis requiring hospitalization, no history of chronic diseases, and no antibiotic or probiotic use within the preceding 3 months. Exclusion criteria encompassed pre-enrollment corticosteroid use; active serious bacterial infection upon admission; chronic liver disease (other etiology that differ from MAFLD: including viruses, and alcohol abuse); pregnancy; and HIV infection.

According the National Institutes of Health (NIH) guidelines [56], COVID-19 severity was categorized into moderate, severe, and critical subgroups.

Presence or absence of MAFLD was established using the following criteria: presence T2DM, overweight/obesity, or demonstrable evidence of metabolic syndrome [57,58]. The hepatic steatosis index (HSI) was employed to evaluate the presence of hepatic steatosis. This scoring system incorporates body mass index, liver enzymes, and presence of diabetes to estimate the likelihood of liver fat accumulation [59].

### 2.2. Laboratory and Clinical Data

During the study, a comprehensive set of laboratory tests were performed as part of the routine clinical workup. This analysis encompassed hematological parameters including white blood cell count with differential, erythrocyte sedimentation rate, hematocrit, and platelet count. Coagulation parameters assessed were international normalized ratio (INR), prothrombin time (PT), activated partial thromboplastin time (aPTT), and fibrinogen. Liver function was evaluated through measurement of total bilirubin, alanine aminotransferase (ALT), and aspartate aminotransferase (AST). Renal function was assessed by measuring creatinine levels. Markers of cholestasis, specifically gamma-glutamyl transferase (GGT), were also included in the laboratory panel. C-reactive protein (CRP) served as an inflammatory marker. Finally, blood glucose levels were measured for all participants. Body mass index (BMI) was documented for each individual.

### 2.3. Gene Expression Analysis

#### 2.3.1. RNA Extraction and cDNA Synthesis

Total RNA was extracted from the collected blood samples using a standard protocol using NucleoZOL (740404.200, Düren, Germany). The extracted RNA was dissolved in RNase-free water to obtain a concentration of 2 µg/µL.

Total RNA was isolated from collected blood samples using a commercially available NucleoZOL reagent (740404.200, Düren, Germany) following a standardized protocol. Extracted RNA was eluted in RNase-free water to achieve a final concentration of 2 µg/µL. Complementary DNA (cDNA) synthesis was subsequently performed using a RevertAid First Strand cDNA Synthesis Kit (K1621, Vilnius, Lithuania) according to the manufacturer’s instructions.

#### 2.3.2. Real-Time PCR Amplification

A Bio-Rad CFX 96 Real-Time PCR Detection System (185-5096, Bio-Rad, Hercules, CA, USA) was employed to quantify the expression levels of four target genes: *AHR*, *FFAR2*, *FXR*, and *TGR5*. Maxima SYBR Green/ROX qPCR Master Mix (2X) (K0221, Thermo Scientific, Wilmington, DE, USA) and gene-specific primers were utilized for the amplification reaction. Each reaction mixture contained 20 µL of nuclease-free water, 0.5 µL of each forward and reverse primer, 2 µL of cDNA template, and 10 µL of 2X Maxima SYBR Green/ROX qPCR Master Mix. The PCR cycling conditions consisted of an initial denaturation step at 95 °C for 10 min, followed by 45 cycles of denaturation at 95 °C for 15 s, primer annealing at 60 °C for 40 s, and extension at 72 °C for 40 s.

The housekeeping gene Glyceraldehyde 3-phosphate dehydrogenase (GAPDH) was selected for normalization of target gene expression levels. The comparative Ct (2-ΔΔCt) method was employed to quantify the relative expression of target genes (*AHR*, *FFAR2*, *FXR*, and *TGR5*) normalized to the housekeeping gene. Ct values were first converted to relative expression values using a formula that compares the Ct value of the target gene to the Ct value of the housekeeping gene. These relative expression values were then transformed into Log2 values using the formula Log2 (relative expression).

Two control groups were employed: a group without COVID-19 for the COVID-19 without MAFLD group and a group with COVID-19 but without MAFLD for the COVID-19 with MAFLD group.

### 2.4. Statistical Analysis

Patient characteristics and clinical data were rigorously assessed and presented using descriptive statistics. The Shapiro–Wilk test was employed to evaluate the normality of the data distribution. Given the absence of normality, medians and interquartile ranges were calculated for all variables to summarize their central tendency and dispersion.

Due to the non-normal distribution of the data, non-parametric statistical tests were utilized for subsequent analyses. The Mann–Whitney U test was implemented for comparisons between two independent groups. For comparisons involving three or more groups, the Kruskal–Wallis test, a non-parametric alternative to one-way ANOVA, was employed. Dunn’s multiple comparison test was then conducted for post hoc pairwise comparisons between groups.

All statistical tests were two-tailed with a significance level set at *p*-value less than 0.05. Spearman’s rank correlation coefficient was calculated to assess the relationships between continuous variables within a correlation matrix. Principal component analysis (PCA) was implemented to identify factors associated with COVID-19 severity and the presence of MAFLD.

Statistical analyses were performed using commercially available software programs, including GraphPad Prism (version 8.4.3) and IBM SPSS Statistics (version 25).

## 3. Results

### 3.1. Comparing Group Expression

Among the 30 patients with COVID-19 included in the study, 15 were classified into the MAFLD group (53.3% male; median age 67 years, IQR 51–78 years). The remaining 15 patients comprised the non-MAFLD group (60.0% male; median age 64 years, IQR 49–72 years). The baseline demographic characteristics were statistically similar between the two groups.

**Figure 1:**
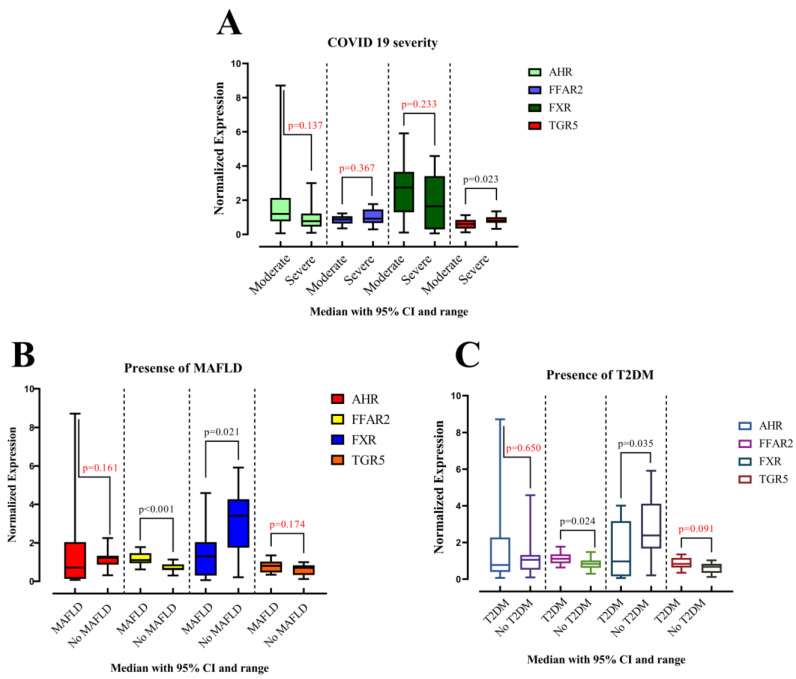
Comparison of the medians of two groups among patients: (**A**) with moderate and severe COVID-19; (**B**) with or without MAFLD; (**C**) with or without T2DM. Data are presented as medians for each group. Statistical comparisons were performed using the Mann–Whitney U test.

Our investigation into gene expression revealed no significant differences attributable to sex (male vs. female). However, compared to the control subjects, individuals with MAFLD displayed a statistically significant decrease in *FXR* expression (*p* = 0.021) and a significant increase in *FFAR2* expression (*p* < 0.001) (Figure 1). The expression levels of *AHR* and *TGR5* were not significantly different between cases and controls.

An analysis of COVID-19 severity revealed significantly lower *TGR5* expression (*p* = 0.023) in patients with severe disease compared to those with moderate illness. The expression of *AHR*, *FFAR2*, and *FXR* did not exhibit statistically significant differences between these groups (Figure 1).

Interestingly, *AHR* expression was significantly lower in subjects with pneumonia (*p* = 0.001) compared to controls. Gene expression levels for all four genes were not significantly different between obese and non-obese subjects. Similarly, no significant differences were observed in gene expression for arterial hypertension or coronary heart disease.

In contrast, individuals with T2DM displayed a significant increase in *FFAR2* expression (*p* = 0.024) and a significant decrease in *FXR* expression (*p* = 0.035) compared to controls (Figure 1). Expression of *AHR* and *TGR5* did not show statistically significant differences in this population. A detailed description of these data is provided in Table 1.

*FFAR2* expression appears to be elevated in MAFLD and T2DM, while *FXR* expression is lowered. *TGR5* expression is reduced in severe COVID-19 compared to moderate cases. *AHR* expression is lower in subjects with pneumonia. No significant associations were found between gene expression and obesity, arterial hypertension, or coronary heart disease.

We further investigated how gene expression levels within the MAFLD group affected clinical outcomes (Table 2). Patients were stratified based on down-regulated, unchanged, or up-regulated expression of specific genes.

In the non-MAFLD group, patients with down-regulated *FXR* gene expression exhibited altered laboratory values upon admission compared to those with unchanged *FXR* expression. They had significantly higher levels of band neutrophils (*p* = 0.045), INR (*p* = 0.038), PT (*p* = 0.021), and GGT (*p* = 0.008). Interestingly, these patients also presented with higher albumin levels at both admission (*p* = 0.045) and discharge (*p* = 0.004).

Conversely, patients with down-regulated *FFAR2* gene expression displayed lower albumin levels at discharge (*p* = 0.018) compared to the unchanged *FFAR2* group.

Within the MAFLD group, patients with up-regulated *FFAR2* expression demonstrated a longer length of hospital stay (*p* = 0.001), higher leukocyte levels on admission (*p* = 0.010), and lower ALP (alkaline phosphatase) levels on admission and discharge (*p* < 0.02) compared to patients with unchanged *FFAR2* expression. Patients with down-regulated *FXR* expression displayed opposite trends: shorter hospital stay (*p* = 0.033) and higher ALP levels on admission (*p* = 0.006) and discharge (*p* = 0.017) compared to those with unchanged *FXR* expression.

Additionally, patients with down-regulated *AHR* expression presented with elevated INR and PT levels upon admission compared to patients with unchanged and up-regulated *AHR* expression (*p*-values < 0.05).

### 3.2. Relative Expression of AHR, FFAR2, FXR, and TGR5 in COVID-19 Patients with and without MAFLD

**Figure 2:**
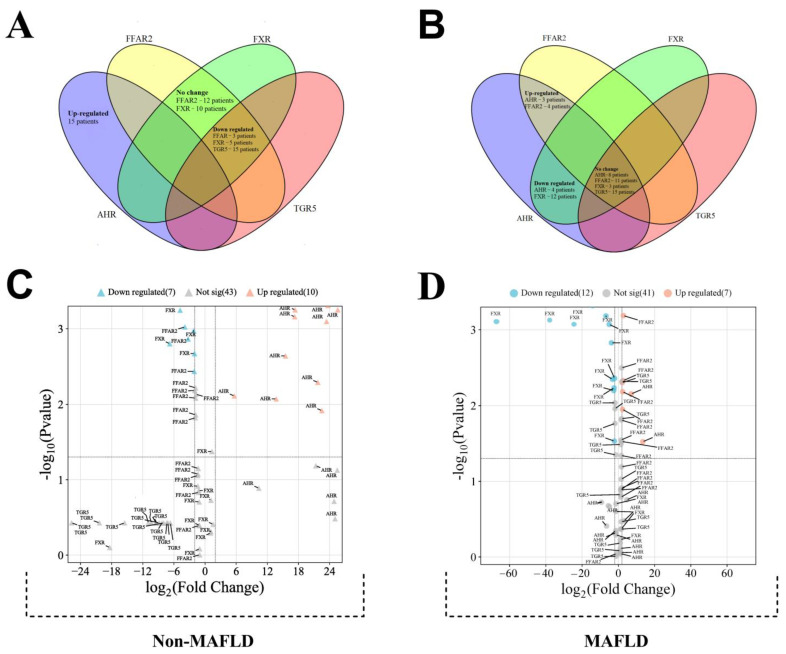
This figure depicts the gene expression profile in patients with COVID-19. Venn diagram illustrating changes in gene regulation for patients with COVID-19 who (**A**) do not have MAFLD; (**B**) have MAFLD. The volcano plot represents statistically significant alterations in the expression levels of the investigated genes (*AHR*, *FFAR2*, *FXR,* and *TGR5*) for the group of fifteen patients with COVID-19 without MAFLD (**C**) and with MAFLD (**D**).

This study investigated the messenger RNA (mRNA) expression levels of four genes (*AHR*, *FFAR2*, *FXR*, and *TGR5*) in patients with COVID-19, stratified by the presence or absence of metabolic-associated fatty liver disease (MAFLD) (Figure 2). To account for potential confounding factors, relative normalized expression was calculated using the PCR method. Two control groups were employed: a group without COVID-19 for the COVID-19 without MAFLD group and a group with COVID-19 but without MAFLD for the COVID-19 with MAFLD group.

An initial analysis of gene expression was conducted in all of the patients with COVID-19 (Figure 2). In the group without MAFLD (Figure 2, Panel A), a distinct pattern emerged. Fifteen patients displayed up-regulation of the *AHR* gene, while *TGR5* was down-regulated in the same patients. Additionally, three and five patients from this group exhibited down-regulation of the *FFAR2* and *FXR* genes, respectively.

Conversely, the group with MAFLD (Figure 2, Panel B) showed a different expression profile. Here, the *FXR* gene was down-regulated in twelve patients, while the *FFAR2* gene was up-regulated in four patients. Interestingly, the *AHR* gene expression was mixed in this group, with up-regulation observed in three patients and down-regulation in four patients.

A volcano plot analysis was employed to further refine the findings by identifying statistically significant changes in gene expression (Figure 2, Panels C and D). This analysis takes into account both the fold-change and statistical significance of gene expression differences.

Within the group without MAFLD, significant differences were observed in a limited number of patients. Ten patients displayed significant up-regulation of *AHR*, while three and four patients showed significant down-regulation of *FFAR2* and *FXR*, respectively.

The MAFLD group exhibited a more distinct expression pattern. Twelve patients displayed significant down-regulation of the *FXR* gene. Only two patients showed significant up-regulation of the *AHR* gene, while four patients had significant up-regulation of the *FFAR2* gene. One patient in the MAFLD group displayed significant up-regulation of the *TGR5* gene.

Patients with COVID-19 exhibited significant changes in gene expression. There was a 21.3-fold up-regulation (CI: 16.0–26.5) of the *AHR* gene, a 1.85-fold down-regulation (CI: 1.42–2.28) of the *FFAR2* gene, and a 15.7-fold down-regulation (CI: 1.42–2.28) of *TGR5* gene.

Furthermore, the presence of MAFLD in patients with COVID-19 resulted in a distinct expression pattern compared to controls without MAFLD. Patients with MAFLD displayed a 1.69-fold up-regulation (CI: 1.21–2.18) of the *FFAR2* gene and an 11.7-fold down-regulation (CI: 1.11–22.2) of the FXR gene compared to the control group.

### 3.3. Correlation Analysis of Genes Normalized Expression

**Figure 3:**
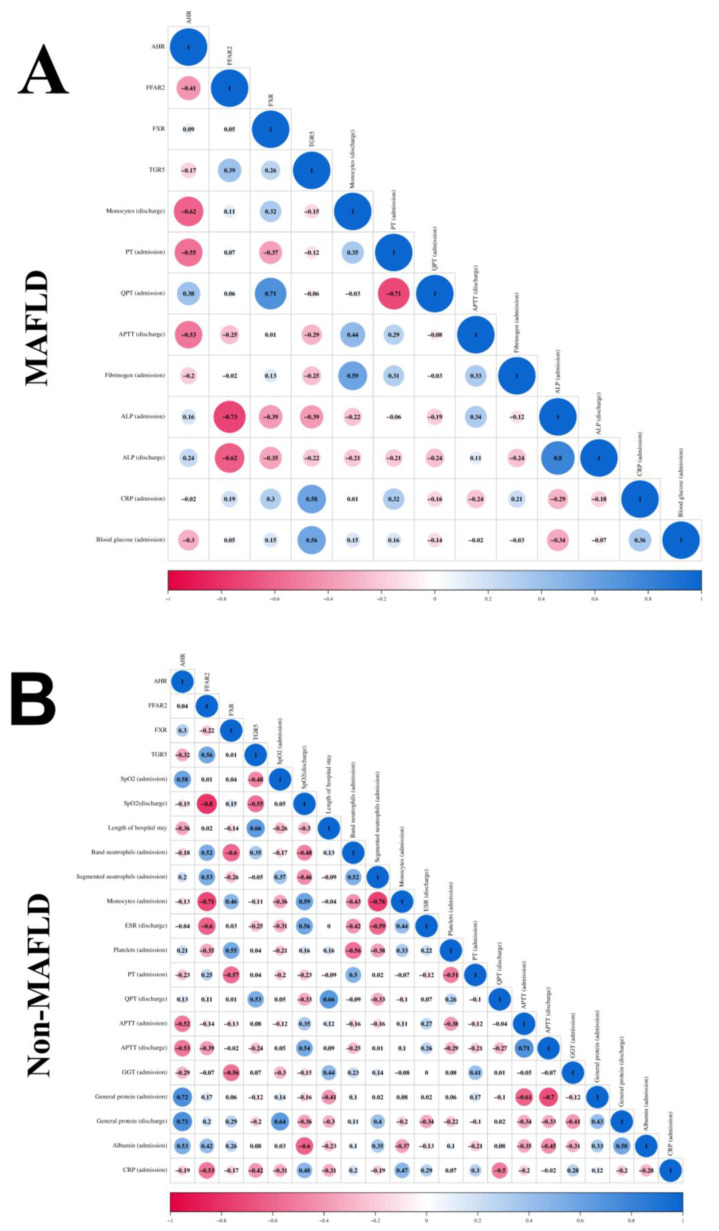
Spearman correlation correlogram used for correlations between continuous data in COVID-19 patients with (**A**) without (**B**) MAFLD. Red: Strong negative correlation (r = −1.0). Blue: Strong positive correlation (r = 1.0).

This section explores the relationships between gene expression levels and various clinical parameters in COVID-19 patients without MAFLD. The analysis employed the Spearman’s rank correlation coefficient (r) to assess the strength and direction of the associations (Figure 3B).

The normalized expression level of the *AHR* gene exhibited positive correlations with several parameters. These included SpO_2_ (admission oxygen saturation, r = 0.58, *p* = 0.001), general protein levels at both admission (r = 0.72, *p* = 0.003) and discharge (r = 0.73, *p* = 0.003), and albumin level at admission (r = 0.53, *p* = 0.043). Conversely, *AHR* expression showed negative correlations with APTT (activated partial thromboplastin time) at both admission (r = −0.52, *p* = 0.049) and discharge (r = −0.53, *p* = 0.042).

The normalized expression level of the *FFAR2* gene displayed a positive correlation with the normalized expression level of the *TGR5* gene (r = 0.56, *p* = 0.032). Additionally, it correlated positively with band neutrophils (r = 0.52, *p* = 0.049) and segmented neutrophils (r = 0.53, *p* = 0.044) at admission. However, *FFAR2* expression exhibited a negative correlation with SpO_2_ at discharge (r = −0.80, *p* = 0.001), monocytes at admission (r = −0.71, *p* = 0.004), and ESR (erythrocyte sedimentation rate) at discharge (r = −0.60, *p* = 0.020).

The normalized expression level of the *FXR* gene showed a positive correlation with platelet level at admission (r = 0.55, *p* = 0.038). Conversely, it exhibited negative correlations with band neutrophils (r = −0.60, *p* = 0.021) at admission, PT (prothrombin time) at admission (r = −0.57, *p* = 0.030), and GGT at admission (r = −0.56, *p* = 0.034).

The normalized expression level of the *TGR5* gene displayed a positive correlation with length of hospital stay (r = 0.66, *p* = 0.009) and QPT level at admission (r = 0.53, *p* = 0.045). However, it showed a negative correlation with SpO_2_ at discharge (r = −0.55, *p* = 0.034).

Next, we analyzed the relationships between gene expression levels and clinical parameters in patients with COVID-19 and MAFLD (Figure 3A).

The normalized expression level of the *AHR* gene exhibited negative correlations with monocyte count at discharge (r = −0.62, *p* = 0.016), PT at admission (r = −0.55, *p* = 0.0437), and APTT at admission (r = −0.53, *p* = 0.044).

The normalized expression level of the *FFAR2* gene displayed a negative correlation with ALP (alkaline phosphatase) levels at both admission (r = −0.73, *p* = 0.003) and discharge (r = −0.62, *p* = 0.016).

The normalized expression level of the *FXR* gene showed a positive correlation with QPT (quick prothrombin time) at admission (r = 0.71, *p* = 0.004).

The normalized expression level of the *TGR5* gene displayed positive correlations with CRP (C-reactive protein) at admission (r = 0.58, *p* = 0.026) and blood glucose levels (r = 0.56, *p* = 0.030).

### 3.4. Principal Component Analysis

**Figure 4:**
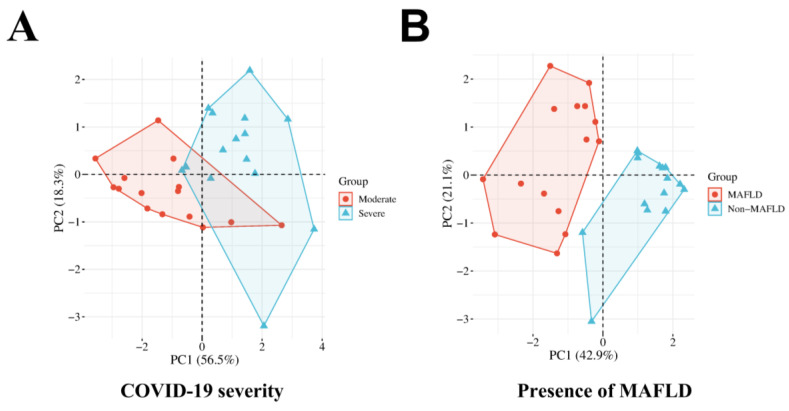
Principal component analysis: (**A**) COVID-19 severity; (**B**) presence of MAFLD.

We employed principal component analysis (PCA) to explore the underlying factors contributing to COVID-19 severity and the presence of MAFLD (Figure 4).

The PCA identified two principal components (PCs) for COVID-19 severity, explaining a total of 74.8% of the variance (56.4% and 18.3% for PC1 and PC2, respectively). The Kaiser–Meyer–Olkin (KMO) measure of sampling adequacy was 0.747, indicating a suitable correlation matrix for PCA. Bartlett’s test of sphericity yielded a significance level of *p* < 0.001, further supporting the applicability of PCA.

PC1 encompassed length of hospital stay, neutrophil-to-lymphocyte ratio (NLR), and lymphocyte count (discharge). PC2 comprised community-acquired pneumonia (CAP) status and oxygen saturation (SpO_2_) upon admission. Segmented neutrophils (discharge) were excluded due to cross-loadings on both PCs, hindering a clear attribution to either component (Table 3).

PCA identified two PCs for the presence of MAFLD, accounting for 64% of the total variance (42.9% and 21.1% for PC1 and PC2, respectively). The KMO measure was 0.588, signifying an adequate correlation matrix for PCA. The Bartlett’s test resulted in a significance level of *p* < 0.001, supporting the use of PCA.

PC1 included body mass index (BMI), normalized expression levels of *FFAR2* and *FXR*, and the presence of T2DM. PC2 constituted of SpO_2_ (admission) and the requirement for supplemental oxygen therapy (Table 4).

## 4. Discussion

Our investigation explored potential associations between gene expression of *AHR*, *FFAR2*, *FXR*, and *TGR5*, and COVID-19 outcomes in patients with MAFLD. Prior research demonstrated that MAFLD is a risk factor for severe COVID-19 presentations and ICU admissions [10,11]. However, the impact of MAFLD on mortality rates remains inconclusive, and explaining their interaction is still quite difficult. Elucidating the interplay between these factors poses a significant challenge. In this context, analyzing gene expression levels holds promise in uncovering novel insights into the combined course of COVID-19 and MAFLD.

Consistent with the *AHR* gene’s known role in regulating immune and inflammatory responses [18], *AHR* expression demonstrated a trend towards down-regulation in patients with severe COVID-19 compared to those with a moderate infection. This observation may be attributed to a potential protective role of *AHR* in mitigating cytokine storm and the pro-inflammatory surge characteristic of septic shock [18].

The role of *AHR* in COVID-19 viral infection was further characterized and proved to be proviral, enhancing viral replication, by Shi et al., 2023 [60]. In line and accordance with this proviral role of *AHR* in COVID-19 infection, we found a 21.3-fold up-regulation of *AHR* observed in COVID-19 patients compared to controls.

In line with previous observations by Lawrence B.P. et al. (2013), the role of *AHR* in infection appears to be context dependent. Their work suggests that *AHR* modulates various regulatory pathways that interact with infection-related signals. These interactions, depending on factors such as the pathogen type and the site of infection, can lead to diverse outcomes [61]. We revealed that *AHR* expression was also lowered in patients with pneumonia (*p* < 0.001), suggesting potential links to impaired local pulmonary immunity and an increased susceptibility to infection. Interestingly, the *AHR* level had positive correlations with the SpO_2_ level at admission in patients with COVID-19.

While *AHR* expression remained largely unchanged in MAFLD patients, those with COVID-19 and MAFLD who displayed down-regulated *AHR* exhibited higher INR and PT levels (coagulation parameters). These findings suggest a potential role for *AHR* in the complex interplay between COVID-19, MAFLD, and coagulopathy. However, further investigation is needed to elucidate the underlying mechanisms and potential therapeutic implications.

The *FFAR2* gene, known to regulate lipid metabolism and glucose levels [31], may influence the course of MAFLD. While not directly expressed in liver cells (hepatocytes), *FFAR2* is present in fat cells (adipocytes) and immune system cells like macrophages and neutrophils [29,30]. *FFAR2* can modulate inflammation through both immune cell activation [31] and gut microbiota interactions [32]. Some studies suggest it can decrease the inflammatory marker IL-6 by regulating beneficial gut bacteria (probiotics) [35,36]. Our findings demonstrate higher normalized *FFAR2* expression in MAFLD patients compared to controls, with similar observations in patients with type 2 diabetes mellitus (T2DM). However, no significant differences were observed between moderate and severe COVID-19 cases.

Within the MAFLD cohort, up-regulated *FFAR2* gene expression was associated with a longer hospital stay, a higher white blood cell (leukocyte) count at admission, and lower alkaline phosphatase (ALP) levels. It is important to note that most patients did not exhibit up-regulated *FFAR2*. In patients with COVID-19 and MAFLD, *FFAR2* expression showed positive correlations with segmented neutrophils and bands (immature neutrophils), indicating a potential influence on specific immune cell populations. Interestingly, negative correlations were observed with monocytes. PCA analysis further identified *FFAR2* as a factor associated with the presence of MAFLD.

Bile acids (BAs) have been proposed to modulate SARS-CoV-2’s entry into host cells through *FXR* and *GPBAR1* by regulating ACE2 expression in various tissues [49]. Additionally, BAs can suppress intestinal dendritic cell (DC) differentiation and activation via *FXR* [62,63], and regulate inflammatory responses through *TGR5* activation, leading to decreased IFN-γ, IL-1β, IL-6, and TNF-α, and increased IL-10 [42]. *FXR* and *TGR5* activation can also improve metabolic health by reducing steatosis (fatty liver) via inhibiting lipogenesis and decreasing hepatic inflammation [52], as well as by mitigating high-fat diet (HFD)-induced glucose intolerance and insulin resistance [53].

Our study revealed significantly lower normalized expression levels of *FXR* in patients with both MAFLD and T2DM, while *TGR5* expression was lower in patients with severe COVID-19 compared to moderate cases. In COVID-19 patients, down-regulated *FXR* expression correlated with higher levels of immature neutrophils (bands), INR (blood clotting parameter), PT (blood clotting parameter), and GGT (liver enzyme) upon admission, alongside a higher albumin level. Interestingly, within the MAFLD cohort, 12 out of 15 patients displayed a substantial (11.7-fold) down-regulation of *FXR*, which was associated with a shorter hospital stay and a higher ALP level (another liver enzyme). Regarding *TGR5*, its normalized expression level in COVID-19 patients showed a positive correlation with hospital stay duration and a negative correlation with SpO_2_ (oxygen saturation) at discharge. Notably, changes in both *FXR* and *TGR5* expression were identified as contributing factors associated with the presence of MAFLD in PCA analysis.

## 5. Limitations

We acknowledge that our study has several limitations. First, the relatively small sample size employed restricts the generalizability of our findings to a broader population. A larger, multicenter study would be necessary to confirm these observations and enhance their generalizability. Second, the monocentric design of this study inherently limits the population studied and may introduce selection bias. Ideally, future studies would incorporate participants from multiple centers to achieve a more representative sample.

This study did not directly compare gene expression in patients infected with COVID-19 to a non-infected control group. Instead, it focused on the differential expression between moderate and severe COVID-19 cases. This limits the generalizability of the findings to the broader spectrum of COVID-19 infection severity.

## 6. Conclusions

This study investigated the relationships between gene expression of *AHR*, *FFAR2*, *FXR*, and *TGR5*, and COVID-19 outcomes in patients with MAFLD. Our findings highlight the potential modulatory roles of these genes, particularly *AHR* and *FXR*, in both COVID-19 severity and MAFLD presentation. We observed an altered expression of *AHR* in patients with COVID-19, potentially influencing immune responses and recovery. In MAFLD, down-regulated *AHR* was associated with increased coagulation parameters. *FFAR2* expression was elevated in MAFLD patients and correlated with specific immune cell populations and hospital stay duration. Interestingly, *FXR* expression was significantly lower in both MAFLD and severe COVID-19, potentially linking it to metabolic health and inflammatory processes. This study contributes to a growing understanding of the complex interplay between gene expression, MAFLD, and COVID-19 outcomes. By identifying potential gene targets, we pave the way for future research that could lead to improved clinical strategies for managing patients with these conditions.

## Figures and Tables

**Table 1:** The expression levels of *AHR*, *FFAR2*, *FXR*, and *TGR5* across various conditions.

		Normalized Expression (Median, IQR ^a^)			
		*AHR*	*FFAR2*	*FXR*	*TGR5*
Sex	Male (*n* = 17)	0.871 (0.519–1.53)	0.938 (0.621–1.14)	1.88 (0.863–3.77)	0.798 (0.404–0.999)
	Female (*n* = 13)	1.22 (0.494–1.72)	0.876 (0.656–1.1)	1.92 (0.430–3.54)	0.681 (0.387–0.848)
	*p*-value ^b^	*p* = 0.837	*p* = 1.000	*p* = 0.650	*p* = 0.509
MAFLD	Presence (*n* = 15)	0.715 (0.133–2.04)	1.11 (0.938–1.46)	1.3 (0.298–2.04)	0.801 (0.46–1.03)
	Absence (*n* = 15)	1.22 (0.863–1.33)	0.646 (0.584–0.88)	3.41 (1.75–4.27)	0.702 (0.325–0.832)
	*p*-value	*p* = 0.161	*p* < 0.001	*p* = 0.021	*p* = 0.174
COVID 19 severity	Moderate (15)	1.20 (0.77–2.14)	0.876 (0.64–1.07)	2.74 (1.30–3.66)	0.606 (0.344–0.846)
	Severe (15)	0.774 (0.456–1.22)	0.928 (0.665–1.46)	1.65 (0.298–3.41)	0.801 (0.681–1.01)
	*p*-value	*p* = 0.137	*p* = 0.367	*p* = 0.233	*p* = 0.023
Presence of pneumonia	Presence (*n* = 16)	0.649 (0.351–1.13)	0.997 (0.7–1.4)	1.7 (0.364–2.87)	0.815 (0.484–1)
	Absence (*n* = 14)	1.53 (0.863–2.44)	0.871 (0.636–0.999)	3.19 (1.04–4.08)	0.65 (0.348–0.812)
	*p*-value	*p* = 0.001	*p* = 0.193	*p* = 0.093	*p* = 0.179
Obesity	Presence (*n* = 10)	0.847 (0.375–2.67)	1.05 (0.868–1.23)	1.86 (0.539–3.17)	0.763 (0.348–1.05)
	Absence (20)	1.04 (0.616–1.32)	0.835 (0.628–1.09)	2.31 (0.707–3.92)	0.703 (0.436–0.856)
	*p*-value	*p* = 0.846	*p* = 0.109	*p* = 0.530	*p* = 0.373
T2DM	Presence (*n* = 10)	0.772 (0.396–2.28)	1.12 (0.868–1.35)	0.971 (0.160–3.17)	0.833 (0.634–1.16)
	Absence (*n* = 20)	1.06 (0.519–1.32)	0.835 (0.620–1.02)	2.39 (1.67–4.12)	0.671 (0.345–0.842)
	*p*-value	*p* = 0.650	*p* = 0.024	*p* = 0.035	*p* = 0.091
Arterial hypertension	Presence (*n* = 20)	0.891 (0.552–1.25)	0.953 (0.7–1.13)	1.98 (0.698–3.5)	0.774 (0.569–0.986)
	Absence (*n* = 10)	1.08 (0.124–1.86)	0.761 (0.614–1.08)	1.47 (0.608–4.48)	0.581 (0.308–0.850)
	*p*-value	*p* = 0.948	*p* = 0.328	*p* = 0.983	*p* = 0.169
Coronary heart disease	Presence (*n* = 13)	0.863 (0.607–1.67)	0.987 (0.754–1.14)	3.14 (0.237–3.77)	0.705 (0.598–1.03)
	Absence (*n* = 17)	0.919 (0.386–1.53)	0.876 (0.601–1.08)	1.80 (0.727–3.54)	0.702 (0.336–0.855)
	*p*-value	*p* = 0.967	*p* = 0.133	*p* = 1.000	*p* = 0.229

^a^ Median and interquartile range (IQR) were used to summarize the data. ^b^ Mann–Whitney test. Ct values were converted to relative expression values. These relative expression values were then transformed into Log2 values (relative expression).

**Table 2:** Difference in laboratory outcomes in patients with down-regulated, no change, and up-regulated gene expression.

No-MAFLD Cohort							
	Regulation of *FXR* gene expression						
	Down regulated (*n* = 5)			No change (*n* = 10)			*p*-value ^a^
Band neutrophils, % (admission)	9 (8.5–18)			6 (4–10)			*p* = 0.045
INR*, n (admission)	1.1 (1.06–1.38)			0.96 (0.865–1.06)			*p* = 0.038
PT*, sec (admission)	14.2 (13.5–16.6)			12.5 (11.4–13.2)			*p* = 0.021
GGT*, unit/L (admission)	67 (49–127)			31(19.1–42.5)			*p* = 0.008
Albumin, g/L (admission)	40 (35–40.5)			45 (40–59.5)			*p* = 0.045
Albumin, g/L (discharge)	37 (33.5–40.5)			47.5 (42.5–50)			*p* = 0.004
	Regulation of *FFAR2* gene expression						
	No change (*n* = 12)			Down regulated (*n* = 3)			*p*-value ^a^
Albumin, g/L (discharge)	46 (41.5–49)			35 (32–40)			*p* = 0.018
**Mafld Cohort**							
	Regulation of *FFAR2* gene expression						
	No change (*n* = 11)			Up regulated (*n* = 4)			*p*-value ^a^
Length of hospital stay, days	11 (10–13)			16.5 (15.3–17)			*p* = 0.001
Leukocytes, 10^9^/L (admission)	7 (6.59–8.97)			11.3 (10.4–14.6)			*p* = 0.010
ALP*, mmol/L (admission)	132 (107–271)			89.5 (80.8–96.8)			*p* = 0.006
ALP, mmol/L (discharge)	132 (106–195)			90.5 (79–112)			*p* = 0.017
	Regulation of *FXR* gene expression						
	Down regulated (*n* = 12)			No change (*n* = 3)			*p*-value ^a^
Length of hospital stay, days	11.5 (10–13.8)			17 (13–17)			*p* = 0.033
ALP, mmol/L (admission)	130 (100–246)			86 (79–103)			*p* = 0.031
	Regulation of *AHR* gene expression						
	Down regulated (*n* = 4)		No change (*n* = 8)		Up regulated (*n* = 3)		*p*-value ^b^
INR, n (admission)	1.17 (1.06–1.66)		0.98 (0.9–1)		0.99 (0.76–1.14)		*p* = 0.018
PT, sec (admission)	14.8 (13.5–19.4)		12.4(11.6–13.1)		12.4 (10.12–14.9)		*p* = 0.033

^a^ Mann–Whitney test; ^b^ Kruskal–Wallis test. INR—International Normalized Ratio; PT—Prothrombin time; GGT—Gamma-glutamyltransferase; ALP—Alkaline phosphatase. Up- and down regulation was determined by comparing the study group with the control.

**Table 3:** Rotated Component Matrix for COVID-19 severity.

	Component	
	Factor 1	Factor 2
Community-acquired pneumonia	0.227	**0.816**
SpO_2_ (admission)	−0.083	**−0.870**
Length of hospital stay (days)	**0.561**	0
Segmented neutrophils (discharge)	0.868	0.411
NLR (discharge)	**0.918**	0.13
Lymphocytes (discharge)	**−0.903**	−0.307

Extraction Method: Principal Component Analysis. Rotation Method: Varimax with Kaiser Normalization (significant loadings in bold). Rotation converged in 3 iterations.

**Table 4:** Rotated Component Matrix for presence of MAFLD.

	Component	
	Factor 1	Factor 2
BMI	**0.724**	0.129
*FFAR2* Normalized Expression	**0.693**	0.233
*FXR* Normalized Expression	**−0.635**	−0.085
SpO_2_ (admission)	−0.18	**−0.914**
T2DM	**0.768**	0.062
The need for oxygen supply	0.13	**0.938**

Extraction Method: Principal Component Analysis. Rotation Method: Varimax with Kaiser Normalization (significant loadings in bold). Rotation converged in 5 iterations.

## Data Availability

Data are contained within the article.
